# EmoWear: Wearable Physiological and Motion Dataset for Emotion Recognition and Context Awareness

**DOI:** 10.1038/s41597-024-03429-3

**Published:** 2024-06-19

**Authors:** Mohammad Hasan Rahmani, Michelle Symons, Omid Sobhani, Rafael Berkvens, Maarten Weyn

**Affiliations:** 1 University of Antwerp - imec, IDLab - Faculty of Applied Engineering, Sint-Pietersvliet 7, Antwerp, 2000 Belgium; 2 Department of Communication Studies, Faculty of Social Sciences, University of Antwerp, Antwerp, 2000 Belgium

**Keywords:** Communication, Psychology, Biomedical engineering, Computer science

## Abstract

The EmoWear dataset provides a bridge to explore Emotion Recognition (ER) via Seismocardiography (SCG), the measurement of small cardio-respiratory induced vibrations on the chest wall through Inertial Measurement Units (IMUs). We recorded Accelerometer (ACC), Gyroscope (GYRO), Electrocardiography (ECG), Blood Volume Pulse (BVP), Respiration (RSP), Electrodermal Activity (EDA), and Skin Temperature (SKT) data from 49 participants who watched validated emotionally stimulating video clips. They self-assessed their emotional valence, arousal, and dominance, as well as extra questions about the video clips. Also, we asked the participants to walk, talk, and drink, so that researchers can detect gait, voice, and swallowing using the same IMU. We demonstrate the effectiveness of emotion stimulation with statistical methods and verify the quality of the collected signals through signal-to-noise ratio and correlation analysis. EmoWear can be used for ER via SCG, ER during gait, multi-modal ER, and the study of IMUs for context-awareness. Targeted contextual information include emotions, gait, voice activity, and drinking, all having the potential to be sensed via a single IMU.

## Background & Summary

Emotions are complex aspects of the human experience^1^ that are recognized, analyzed, and comprehended in the field of Affective Computing (AC)^2^. Recent research in AC has been frequently addressing influence of context in which emotions arise as a determining factor^3^. Context has a broad definition covering any information that can be used to characterize an individual’s situation^4^, including their physical or physiological signs as well as their relationship with the environment, other individuals, and daily activities^3^. While context can trigger and shape emotional responses^5^, emotions themselves contribute to context^6^ forming a bidirectional relationship. Emotion- and context-awareness enhance the ability of pervasive computing to interact more effectively with humans^7^, spanning a wide range of applications from entertainment^8^ and marketing^9^ to education^10^ and healthcare^11^. In order to benefit from emotion- and context-awareness, their measurement is a key component.

The bidirectional relationship between emotions and context necessitates their concurrent measurement. Measuring multiple aspects of context, from internal, such as emotion or physiological state, to external, such as activity or social context, requires employment of a variety of sensors. Existing technologies allow emotion recognition using physiological^12^, vision^13^, or audio^14^ sensors, among others. Depending on which additional aspects of context are targeted, further environmental^15^, motion^16^, or localization^17^ sensors may be used. However, the use of multiple sensors flags issues such as power consumption, privacy, user discomfort, and high costs^18–20^. A workaround is to restrict sources of context by using only limited number of sensors^18^; although this might come at the expense of constraining the ultimate goal of context-awareness. Such expense can be mitigated by doing more with less and delving into the opportunities of using a single sensor for multiple purposes. Here, our focus is on miniaturized Inertial Measurement Units (IMUs) that are placed on the chest as they provide a unique opportunity to concurrently capture not only movements of the wearer, but also heart- and respiration-originated vibrations, i.e. Seismocardiography (SCG)^21^.

At the same time, the use of Machine Learning (ML) algorithms has made it approachable for Artificial Intelligence (AI) to recognize human emotions through physiological data^12^. Several datasets have been published to address physiological responses to emotional stimuli, *e.g*., EMOGNITION^22^, POPANE^23^, BIRAFFE(2)^24,25^, CASE^26^, ASCERTAIN^27^, DECAF^28^, DEAP^29^, and MAHNOB-HCI^30^. Among the physiological data, Heart Rate Variability (HRV) has been investigated several times for AC with heart-originated biosignals being derived from Electrocardiography (ECG)^31^, Blood Volume Pulse (BVP)^32,33^, and/or Radio Frequency (RF) sensors^34^. However, the potential use of SCG for AC remains untouched. Chest-worn IMUs can provide SCG data as a potential proxy for AC. In addition to SCG, chest-worn IMUs have applications in activity recognition, posture analysis, localization, swallow detection, and Voice Activity Detection (VAD)^21^. With the variety of their offered contextual information, they have the potential to bring high throughput with less cost and power consumption comparing to when multiple sensors are used. Given the role of IMUs in capturing movement-related context, their integration into AC applications can ultimately broaden the spectrum of measured context using the same sensor^35^. This could additionally yield valuable insights into the interplay between physiological responses and body movements during emotional experiences, potentially paving the way for more comprehensive and accurate AC systems. However, existing datasets lack the setup to unveil the potential of the chest-worn IMUs for capturing multiple contextual information with one sensor. In this paper, we describe our dataset called EmoWear, with which we try to fill mentioned gaps by touching upon emotions, activity, swallow, and voice activity, as targeted contextual aspects.

Two key enablers distinguish EmoWear from existing datasets in AC: first, the inclusion of SCG extracted from either Accelerometer (ACC) or Gyroscope (GYRO) modalities, which we validated with physiological sensor data; and second, the incorporation of concurrent IMU-oriented contextual tasks (detecting gait, voice activity, and drinking), alongside the standard setup for emotion recognition. Among AC datasets that incorporate IMU sensor data, none are made for investigation of SCG in the field of AC. BIRAFFE2^25^ offers ACC data collected from a gamepad; K-EmoCon^36^, K-EmoPhone^2^, and Schmidt *et al*.^32^ collect their ACC data only from the wrist. Only WESAD^37^ and cStress^38^ provide chest-sensed acceleration data. However, cStress^38^ has a sampling rate of 16 Hz for its ACC data, which is not suitable for SCG^21^, nor does it make the data publicly available. While the ACC data provided in WESAD may have utility for SCG, it does not validate usefulness of their ACC data for SCG e.g. by showing its correlation with heart and breathing signals. Moreover, the WESAD dataset differs significantly from ours in that it does not involve contextual tasks that are motion-detectable (such as gait, voice activity, and drinking), focuses solely on amusement and stressful states in its presented stimuli, and notably does not measure GYRO data.

The EmoWear dataset contains around 70 hours of inertial and physiological recordings from 49 adults who have undergone a set of experiments in a lab environment. These experiments include watching 38 emotionally eliciting video clips, self-assessing emotional state, walking a predefined route, reading out sentences, and occasionally drinking water. To quantify emotional states, we employed the widely recognized circumplex model of affects^39,40^, with the valence, arousal, and dominance scales. We included supplementary questions to assess participants’ familiarity with the stimuli and their subjective liking of the presented content. Physiological data was recorded using two consumer-grade wearables: Empatica E4 providing BVP, Electrodermal Activity (EDA), Skin Temperature (SKT), Inter-beat Interval (IBI), and ACC data and Zephyr BioHarness (BH3) providing ECG, Respiration (RSP), SKT, and ACC data. Motion data was exclusively recorded using three ST SensorTile.box (STb) devices, each providing three sources of ACC and one GYRO data. Two of these devices were worn on the bottom of the sternum (used for SCG) and its corresponding height on the subjects’ backs, while the third was attached to a cup of drinking water to capture any drinking activity. Table 1 provides a summary of the main dataset characteristics.

**Table 1:** Summary of the EmoWear dataset characteristics.

Characteristic	Information
Participants	49 (21 females, 27 males, 1 missing data); aged between 21–45 (mean = 29.27, SD = 4.53)
Sensors	ST SensorTile.box (×3), Zephyr BioHarness 3 (×1), Empatica E4 (×1)
Recorded signals	triaxial ACC (×11), triaxial GYRO (×3), ECG (×1), BVP (×1), RSP (×1), EDA (×1), SKT (×1)
Total phases	2 (phase 1: voice activity recording; phase 2: elicit-assess-walk cycles)
Stimuli	38 one-minute-long video clips (HAHV × 10, LAHV × 9, LALV × 10, HALV × 9)
Rating scales	Valence, Arousal, Dominance, Liking, Familiarity
Total duration	69 h 11 min (average per participant: 1 h 26 min)

Acronyms used in the table are: SD = Standard Deviation, ACC = Accelerometer, GYRO = Gyroscope, ECG = Electrocardiography, BVP = Blood Volume Pulse, RSP = Respiration, EDA = Electrodermal Activity, SKT = Skin Temperature, HAHV = High Arousal High Valence, LAHV = Low Arousal High Valence, LALV = Low Arousal Low Valence, HALV = High Arousal Low Valence.

The EmoWear dataset presents the opportunity for combined research, unlocking the potential of a single chest-worn IMU for measuring multiple contextual dimensions it has to offer. Use of duplicate modalities capturing heart activity (i.e., ECG and BVP), allows for the validation of SCG against them for HRV monitoring. Employment of chest-worn IMUs, measuring movement data in both stationary and walking conditions allows for the study of both SCG and gait analysis for Emotion Recognition (ER). Additionally, VAD, drinking, and gait detection are possible using the dataset to discover the possibility of a wider context measurement via the same setup; a selection that expands concurrent applications of chest-worn IMUs. To the best of our knowledge, this is the first dataset to enable investigation of SCG for emotion recognition; and to enable investigating applicability of a single IMU for multiple contextual aspects. Potential research topics that may be investigated using the EmoWear dataset include but are not limited to: (1) use of SCG for AC, (2) emotion recognition in walking, (3) effect of variations of heart rate due to walking on emotion recognition, (4) multi-modal emotion recognition, (5) VAD using chest-worn IMU, (6) swallow detection using chest-worn IMU.

## Methods

### Ethics statement

All the hardware, methods, and procedures associated with the collection of the EmoWear dataset were reviewed in advance by the independent Ethics Committee for the Social Sciences and Humanities of the University of Antwerp, under the file *SHW_22_035*. The committee issued a positive decision regarding the ethical clearance after reviewing the following documents: (1) application file for ethical clearance of the Ethics Committee for the Social Sciences and Humanities of the University of Antwerp, (2) methodology of the study, (3) information sheet for the participant, (4) consent form for the participant, (5) a list of ethical committees to which the research proposal will be presented, (6) all information that will be used to contact the participants, (7) all the diaries or surveys that will be presented to the participants. The participants were informed in advance on the experiment details both orally and in the written form. They were explicitly asked for their consent to participate, and they were informed about their right of withdrawal from the experiments at any stage of the data collection procedure. No personal information that could lead to their identification was saved from the participants.

### Participants

Participants were recruited through advertisements on the following social media platforms: Facebook, Instagram, X (at the time known as Twitter), and LinkedIn. The advertisement invites healthy adults, aged between 18 and 65, without major known heart, movement, or psychological issues, to schedule an appointment for a voluntary data collection session. It was presented in both written form and with graphics, informing participants that they would watch videos while wearing physiological sensors, assess their emotional states, and enjoy a drink afterwards. Additionally, it indicated that the experiments would take approximately two hours of their time.

Out of all those who scheduled appointments, 49 attended their designated slots. None of the attendees were excluded due to health issues; instead, they were asked to disclose any physical or mental disorders in a pre-experiment questionnaire. Each participant is identified by a unique 4-character ID assigned by the Graphical User Interface (GUI), as well as a sequential integer code ranging from 1 to 49. Due to an unintended failure to run the logging software, data from one participant (code: 35, ID: 9W29) were not recorded, reducing the total number of EmoWear participants to 48 (21 females, 27 males) aged between 21 and 45 (mean = 29.27, SD = 4.53).

### Hardware

We used the following wearable sensors to collect inertial and physiological data from the participants: (1) **ST STb** set up to collect acceleration and rotation data from three different IMUs it has: LIS2DW12, LIS3DHH, and LSM6DSOX. Three STbs were employed. One was positioned vertically on the sternum, aligning the bottom of the box (which houses the micro-USB port) with the bottom of the xiphoid process, and the back of the box (which displays the printed device label) was pressed against the skin using an elastic strap for male participants and their own bra for female participants. The second box was placed at the same level as the first, on the subject’s back, secured with the same strap/bra, with the bottom of the box facing the ground and the back against the skin. The third box was attached vertically in a similar orientation to a cup of water on the participant’s desk, using a pair of adhesive Velcro strips. (2) **Zephyr BH3** device, programmatically configured to be worn on the left side, with its log format set to “Enhanced Summary + Wave.” This device logged ACC, ECG, R wave to R wave (RR), raw RSP, and Breath to Breath (BB) data. It was worn immediately below the strap/bra used to secure the STbs. (3) **Empatica E4** wristband worn on the non-dominant hand (as in^37^) to minimize motion artifacts in the data collected from the participants. It recorded various data including BVP, EDA, SKT, Heart Rate (HR), IBI, and ACC.

We selected the STb device because it offers a variety of accelerometers and allows for different configurations of its accelerometers. We employ all of them to provide redundancy, and set them up differently (Table 2) to ensure robust motion sensing on a range of small to big movements. The STb has been previously validated for various applications, including activity recognition^41^, micro-positioning^42^, fall detection^43^, and virtual reality^44^. Prior to our selection, we conducted a literature review on the IMUs used for SCG^21^ and ensured that the available setups of the STb met similar specifications.

**Table 2:** Sensors used in the dataset, their position, provided data, and (configured) setup.

Device	Position(s)	Data	Setup
ST SensorTile.box (STb)	Sternum, Back, Cup-side	Accelerometer: LIS2DW12 (*ACC*_1_)	1600 Hz, ±2 g
		Accelerometer: LIS3DHH (*ACC*_2_)	1100 Hz, ±2.5 g
		Accelerometer: LSM6DSOX (*ACC*_3_)	208 Hz, ±2 g
		Gyroscope: LSM6DSOX (*GY RO*)	208 Hz, ±250 dps
Zephyr BioHarness 3 (BH3)	Chest (below STb strap)	Electrocardiography (ECG)	250 Hz
		Respiration (RSP)	25 Hz
		Accelerometer (ACC)	100 Hz
		R wave to R wave	Variable
		Breath to Breath	Variable
		Breath Rate	1 Hz
		Heart Rate	1 Hz
Empatica E4	Wrist (non-dominant hand)	Blood Volume Pulse (BVP)	64 Hz
		Accelerometer (ACC)	32 Hz
		Electrodermal Activity (EDA)	4 Hz
		Skin Temperature (SKT)	4 Hz
		Heart Rate	1 Hz
		Inter-beat Interval	Variable

“Hz” stands for Hertz, “g” is equal to the gravitational acceleration on Earth’s surface (9.81 *m*/*s*^2^), and “dps” stands for degrees per second.

The Zephyr BioHarness was originally designed for sports performance monitoring and training optimization^45^. Nevertheless, its measurements have been validated for various applications, including ER^46,47^, stress detection^47,48^, energy expenditure estimation^49^, and psychometric research^50^, among others. The measurements of both HR and respiratory rate from this device have demonstrated promising correlation with other laboratory-grade and previously validated portable devices^51^. Presence of this device in our dataset provides additional gateways to validate our SCG signal with the ECG and the respiratory signal of the BH3 device.

Empatica E4 has been previously validated for ER^22,32,52^, stress detection^37,53^, mental fatigue modeling^54^, and energy expenditure estimation^49^ among others. In our dataset, we employed the Empatica E4 to incorporate an additional proxy for the validation of heart monitoring i.e. the BVP, as well as the EDA and the SKT to enable multi-modal ER.

**Fig. 1:**
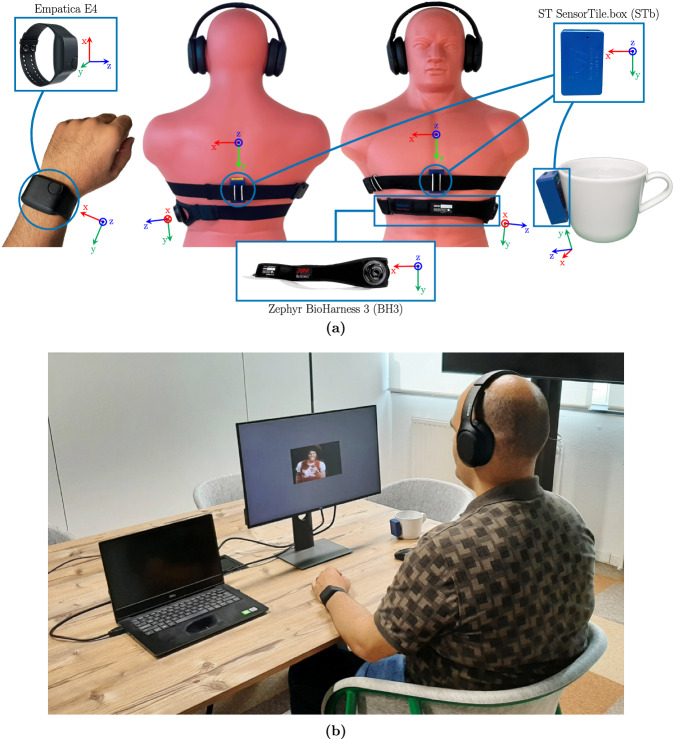
(**a)** Sensor setup used in the dataset. Wearable hardware included: two ST SensorTile.boxes secured with elastic strap, Zephyr BioHarness 3 strap worn on left body side, and Empatica E4 wristband, all having contact with the body skin. An additional ST SensorTile.box was connected to a cup of water. Arrows indicate the positive direction of the Cartesian coordinate system for each sensor, based on which acceleration data is reported in the mat and csv packages. Reprinted with permission from IEEE^55^, slightly modified afterwards. (**b)** An author demonstrates the complete experimental setup in action.

All the wearable sensors were configured to record data offline, storing it within their internal memory. These sensors offered a range of sampling rates for different types of data, which are detailed in Table 2. Fig. 1 illustrates the hardware setup in action. The coordinate systems of all devices, particularly those of the accelerometers, are illustrated in this figure. In order to match coordinate system of the trunk-worn devices, we applied adjustments to the STb accelerometers and gyroscopes. Specifically, we negated the x-axis of the STb accelerometers and gyroscopes. Additionally, we noticed an inconsistency in the y-axis of the STb gyroscopes, where its positive direction was linked with clockwise rotation around its corresponding accelerometer axis. To rectify this, we negated the y-axis of the STb gyroscopes as well. These adjustments are reflected in the processed dataset packages, namely the mat and csv packages, which are explained under Data Records. Directions in the figure reflect the resulting coordinate systems of the accelerometers after these adjustments. Note that all gyroscope axes now have their positive direction on counterclockwise rotation around their corresponding accelerometer axes.

We used a 24-inch Dell U2419H monitor to display our GUI to the participants. Additionally, we employed noise-canceling wireless headphones, specifically the Sony WH-H910N, to deliver audio of the video clips and minimize potential environmental noise disturbances.

### Graphical user interface

We used ColEmo data collection GUI, an open-source software interface that we designed for our study^55^. ColEmo was used to: (1) Present and collect pre-experiment questionnaire, (2) Facilitate experiment phase control by providing timely instructions for every step of the data collection process, (3) Facilitate recording, communication, and verification of vocal vibration data, (4) Presenting emotional stimuli, and (5) Collecting self-assessment records.

ColEmo was run on the participant’s laptop and used the following I/Os to interact with participants: (1) An external monitor to present the software and the stimuli, (2) An external headphone to present the stimuli audio, (3) The laptop’s built-in keyboard for the pre-experiment questionnaire, (4) An external mouse to enter self-assessment reports and press software buttons, (5) The laptop’s built-in microphone to record their temporary voice in the VAD data phase.

#### GUI logs

ColEmo published its logs using the Message Queuing Telemetry Transport (MQTT) protocol, with the participant’s laptop itself serving as the MQTT broker. The logs involved participant’s answers to the questionnaire and the self-assessment reports, as well as event markers that indicated occurrence time for every single phase change of the GUI (*e.g*., beginning of a stimulus video; see Table 7). An MQTT logger application (https://github.com/curzon01/mqtt2sql) installed on the same laptop captured and saved these messages into an SQLite^56^ database. The experimenter used an additional laptop outside the experimental room to monitor the progression of the experiment. Experimenter’s laptop subscribed to all topics published by ColEmo, resulting in the logging messages being captured and displayed on the experimenter’s laptop. Table 3 explains the logging information generated by ColEmo. The topic IDs can be used to find specific types of messages from the SQLite database.

**Table 3:** Description of the GUI logs.

Topic ID	Description	Format	Frequency
239	Participant’s answers to the pre-experiment questionnaire	json	1 per participant
249	Participant’s answers to the self-assessment surveys	json	1 per experiment
237	Event time markers	json	1 per event

The ColEmo app broadcasted these logging information instantly with the MQTT topic IDs mentioned in the table.

### Pre-experiment questionnaire

After obtaining their consent, the participants were asked to complete an electronic pre-experiment questionnaire regarding their long- and short-term conditions prior to the experiments. Such data will help future data users draw their own inclusion criteria based on their specific research requirements. Participants were free to choose the language of the questionnaire, with options including English (default) and Dutch (local language). The questionnaire covered participants’ demographic information, including gender, birth year, education level, dominant hand, vision status, and use of vision aids. Additionally, it inquired about alertness level, normal nightly sleep hours, last night’s sleep duration, any physical or psychological disorders, and the consumption of coffee, tea, alcohol, tobacco, and drugs. Participants were asked to specify the regularity of their substance intake, with separate questions addressing the period up to one day prior to the experiments. These questions were similar to those found in the DEAP dataset^29^, eliminating Electroencephalography (EEG)-related questions. While filling out the questionnaire, participants could ask the experimenter any questions they had for clarification.

### Stimuli

We utilized the same stimuli as those collected and evaluated in the benchmark DEAP dataset^29^. The stimulus selection process in the DEAP dataset involved multiple stages. Initially, 120 stimuli were chosen, with half of them selected through a semi-automatic process and the remaining half chosen manually. Following that, a one-minute highlight segment was designated for each stimulus. Subsequently, an online subjective assessment experiment was conducted based on the well-known Self-Assessment Manikins (SAM) scale^57^. This assessment involved rating the videos according to their emotional valence, arousal, and dominance. The valence-arousal space was divided into four quadrants: high arousal-high valence (HAHV), low arousal-high valence (LAHV), low arousal-low valence (LALV), and high arousal-low valence (HALV). Based on the online assessment, the 10 most effective videos from each quadrant were selected, resulting in a final set of 40 stimuli. We refer to each of the four mentioned quadrants as (reference) labels for the used video clips.

We were unable to locate 2 out of the 40 labeled DEAP stimuli, resulting in a total of 38 stimuli for our dataset. These stimuli were distributed as follows: 10 with the HAHV label, 9 with the LAHV label, 10 with the LALV label, and 9 with the HALV label. The 2 missing stimuli corresponded to experiments with IDs 16 and 35 from the DEAP dataset. The 38 stimuli were presented in the form of 38 experiment cycles, during which the stimulus contents were displayed on the 24-inch monitor. The screen resolution was 1920 × 1080, and the videos were configured to play with a height of 400 pixels while maintaining their original aspect ratio. To minimize bias, the order of the experiments was randomized, following a similar approach to previous studies^26,29^. To ensure that participants concluded the data collection session with a positive experience, the last experiment was always selected among the videos with high labeled emotional valence. To implement this, the last experiment was chosen first and appended to the end of a shuffled list containing the remaining experiments.

### Self-assessment surveys

Annotating emotional state depends on the choice of the emotional model. We used a dimensional approach where participants assessed their own affective state based on the circumplex model of affect, as frequently used by the existing benchmark datasets^29,30,37,58^. In order to facilitate comparison with the DEAP dataset which shares the same stimuli content, we employed the same continuous scales. Participants were instructed to assess their affective state after the presentation of each stimulus video. Same SAMs as in the DEAP dataset were used to assess the emotional valence, arousal, dominance, and liking. The fifth question assessed the participant’s familiarity with the presented music video clip on a discrete scale of 1 (totally new) to 5 (well known). Participants could select their desired value by moving a slider below each question with the external mouse. Table 4 provides the specifications of the content presented to participants for their self-assessments following each stimulus video.

**Table 4:** Content and specifications of the self-assessment survey filled out by the participants after each stimulus presentation.

Scale	Continuity	Range	Presented question
Valence	Continuous	[1,9]	
Arousal	Continuous	[1,9]	
Dominance	Continuous	[1,9]	
Liking	Continuous	[1,9]	
Familiarity	Discrete integers	{1,2,3,4,5}	

### Experimental procedure

The experiments were conducted in a dedicated meeting room within our institute. The experimental room had a rectangular shape measuring approximately 6 *m* × 4 *m*. In the center of the room, there was a rectangular desk measuring 3.6 *m* × 1.2 *m*. Participants were seated at one corner of the desk to perform the experiments.

Upon arrival, participants received a comprehensive briefing regarding the purpose, scope, and details of the experiments. They were explicitly informed of their right to withdraw from the study at any point during the session. Any queries or uncertainties were addressed, and all necessary information was provided in written form. After obtaining explicit consent to participate, participants were given the opportunity to use the restroom before starting the experiments. Additionally, they were kindly requested to mute any electronic devices such as smartphones or smartwatches.

Subsequently, participants completed an electronic pre-experiment questionnaire in the ColEmo software. Meanwhile, the experimenter initiated the wearable devices. The experimenter then assisted participants in wearing the sensors. To facilitate synchronizing the sensors, participants were asked to perform three consecutive jumps with around one second pause in between the jumps. During these jumps, the participant was holding the third STb sensor in their hand. The experimenter pressed a button in ColEmo software, right after the participant’s third jump to indicate that the synchronization jumps were over. The moment of this button press was recorded in the GUI logs.

#### Phase 1: vocal vibration recording

The initial phase of data collection involved recording vocal vibration data with the intention of future IMU data processing for VAD. Participants had the option to decline vocal recording in the ColEmo software if they preferred. This phase was implemented at a later update to the experiment procedure, therefore not all participants have their VAD data recorded (see Usage Notes).

During this phase, participants read out 10 sentences while all wearable measurement hardware was operational. Each sentence was presented to participants for 6 seconds, along with a progress bar that indicated the elapsed time. Participants were asked to read out the sentences within the presentation time for each sentence. It was not possible to proceed to the next sentence if they read too quickly. Two 6-second periods of silence were included before and after the set of all 10 sentences.

**Fig. 2:**
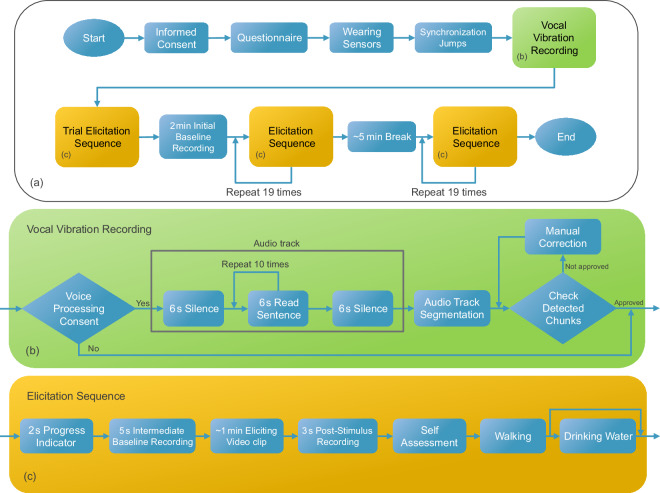
Experiment procedure of the data collection sessions. (**a)** The overall flow. (**b)** Details of the vocal vibration recording (phase 1). (**c)** Details of the elicit-assess-walk sequences in phase 2.

Participants were instructed to place their arms on the desk and maintain a steady position throughout the recording. Simultaneously, the readings were temporarily captured using the laptop’s built-in microphone and subsequently processed to mark the beginning and end of each sentence. To achieve this, we used a Python interface to the WebRTC voice activity detector (https://github.com/wiseman/py-webrtcvad). The Python script processed the microphone recording to generate an initial set of time markers denoting the start and end points of speech segments. These markers were used to segment the audio into discrete chunks, which were then reviewed by the experimenter to verify marker accuracy. If any markers were found to be inaccurately detected, the experimenter had the opportunity to make necessary manual adjustments. Once the correctness of the detected voice chunks was confirmed, the microphone recording was permanently deleted to maintain privacy and confidentiality. An overview of the mentioned procedure is illustrated in Fig. 2b.

The sentences used during this phase were selected from the Common Voice dataset^59^. To ensure ease of reading, our selection criteria included sentences with a length ranging from 6 to 11 words. Moreover, some sentences were slightly simplified in terms of both length and vocabulary. In order to cover a range of sentence types, we included declarative, imperative, and interrogative sentences. These sentences were all presented in English. For reference, Table 5 provides the exact sentences that participants read out during this phase, in the order they were presented to the participants.

**Table 5:** Sentences that participants read out during the phase 1 of the experiments (vocal vibration recording), in the presented order.

#	Type	Sentence
1	Declarative	“Plastic bags turned out to be a problem for the environment.”
2	Declarative	“The wise man may change his opinion, but the fool, never.”
3	Imperative	“Just give me one more minute.”
4	Declarative	“Separation of garbage makes recycling possible.”
5	Imperative	“Please do not feed the ducks!”
6	Declarative	“On warm days and when doing sports you should drink more.”
7	Declarative	“History teaches us that humans do not learn from history.”
8	Interrogative	“How do we know it’s really over?”
9	Interrogative	“Can I get you a drink?”
10	Declarative	“You cannot brew tea in a cold pot.”

#### Phase 2: elicit-assess-walk cycles

This phase forms the main and the lengthiest part of the dataset involving a 2-minute initial baseline recording while presenting a fixation cross on a white screen, and 38 cycles of elicit-assess-walk experiments. Each cycle consisted of the 7 following stages: (1) 2 seconds presentation of the sequence number, to indicate participant’s progress out of the total 38 experiments; (2) 5 seconds presentation of a fixation cross on a white screen, to provide intermediate baselines; (3) presentation of an eliciting stimulus video with around 1-minute length; (4) 3 seconds presentation of the same fixation cross on a white screen; (5) filling out the self-assessment survey with 5 scales (valence, arousal, dominance, liking, and familiarity); (6) walking a fixed certain route and returning to the chair; (7) optionally, drinking water before proceeding to the next experiment. The main outline of the methodology above, including the presentation order and time of each component, the stimuli used, and the provided survey questions, matches that of the benchmark DEAP dataset^29^, with the extension of walking and drinking tasks.

To familiarize participants with the procedure, a trial sequence of the elicit-assess-walk cycle mirroring the main experiment cycles was presented to the participant. The experimenter explained to the participant at each step of the trial sequence, what they had to expect at the real experiments. The trial sequence included an example brief video of a bee on a flower. Participants were informed that the presented video was only a dummy one, and that during the main experiments, they would encounter emotional videos lasting approximately one minute. An example self-assessment survey was also presented, with the experimenter clarifying the meaning of each scale.

During the trial sequence, participants were also informed that they would be asked to stand up and walk after completing the self-assessment survey. The experimenter demonstrated the designated walking route for participants to follow at the end of each experiment cycle. This path covered a total distance of approximately 18 to 19 *m*, involving circling around three sides of the desk, specifically, one long side, one short side, and finally another long side, before returning along the same route. During the walk, an on-screen timer counted 19 seconds before the participants could proceed to the next experiment. Participants were explicitly instructed to walk at their own pace and disregard the on-screen timer.

Purpose of adding such walking stage was multi-fold: First, to enable research on any potential effect of emotional state on gait (as in^35^). Second, to enable research on the effect of physiological parameter changes rising from movement on falsified emotion classification (as in^60,61^). Third, to enable research on movement artifact removal from the SCG signal, either by pure signal processing methods (as in^62,63^), or by using the additional STb on the back (as in^64^). Finally, to possibly facilitate washing out the previous elicited emotion and prepare participant for the next experiment.

After returning, participants were instructed to press the *“continue”* button on ColEmo when they were ready for the next experiment. Additionally, a cup of water, equipped with stb, was provided on the participant’s desk. They were encouraged to drink as needed, but only after completing each walk and before continuing to the next experiment in ColEmo.

At this stage, the experimenter exited the room. Participants initiated the second phase of the experiments, starting with a 2-minute initial baseline recording while a fixation cross was displayed on the screen. The explained experimental sequence was then repeated 19 times, followed by about 5 minutes break. The experimenter returned to the room during the break, offering participants non-alcoholic and non-caffeinated beverages. Once participants were ready, the experimenter exited, allowing participants to complete the remaining 19 experiments.

Upon concluding all experiments, the experimenter re-entered the room to stop the wearable sensors. Total data recording session lasted approximately 1 h 30 min. Participants were then sincerely thanked and offered a drink at our cafe. Fig. 2 shows the detailed flow of the data collection procedure explained above.

### Data cleaning and synchronization

After completing the data collection session, the raw data from all wearable devices was copied to a secure drive. After the whole dataset was collected, we imported the entire dataset into MATLAB to achieve uniform data structures and synchronize the data for all participants. Data cleaning and synchronization was performed with MATLAB R2023b.

**Fig. 3:**
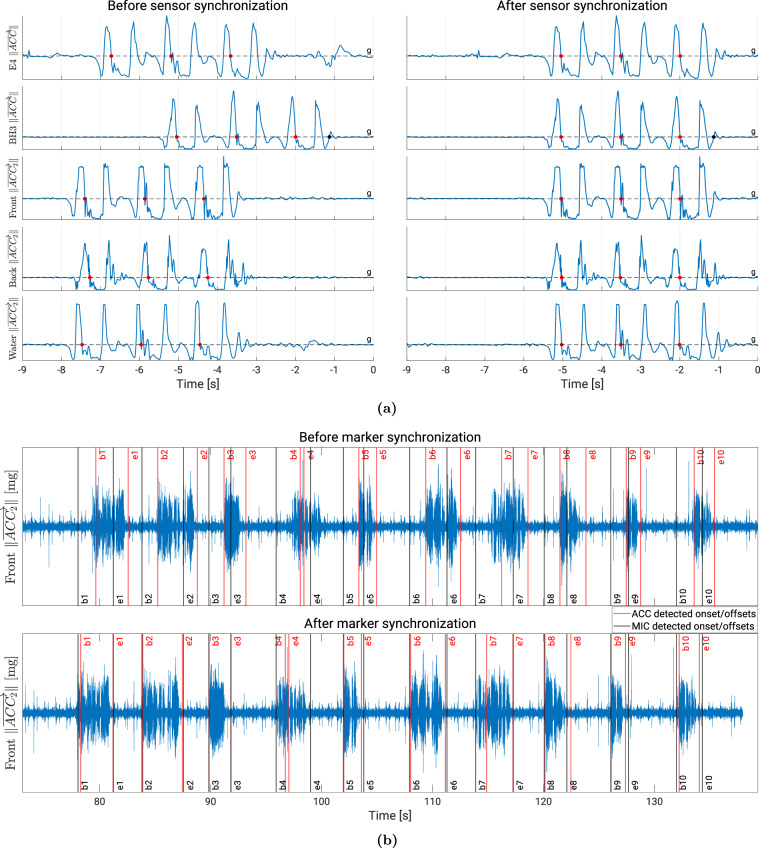
(**a)** Synchronization process of the five sensors using three jumps. Red dots represent the detected top of jump moments where acceleration crosses the gravitational value (g), while black dots denote the specified end of all jumps (only specified on the  BioHarness 3 (BH3) plots). All sensors were synchronized with respect to the Zephyr BH3 device. (**b)** Synchronization of the Front ST SensorTile.box (STb) sensor with the event time markers using voice activity data. Black lines indicate detected voice onsets (*b*#) and offsets (*e*#) using microphone (MIC) found in the event time markers, while red lines represent detected voice onsets and offsets using accelerometer (ACC) data from the Front sensor. The necessary time shift is determined by minimizing the distance between the two sets of markers and is applied to all sensor data to synchronize them with the event time markers.

The synchronization process consisted of two main steps: synchronization of data from different wearable devices, and synchronization of those data with the event time markers generated by ColEmo (see GUI section). For the first part, initially, we processed the three jumps executed by the participants. We identified the moments when each participant reached the apex of their jumps for all devices, resulting in five vectors of three points. These vectors were used to synchronize all sensors with respect to the BH3 device. This synchronization involved minimizing the distance between the vectors of each sensor and that of the BH3 device (see Fig. 3a). Additionally, we compensated for potential clock drift among the sensors by manually identifying corresponding data points among the sensors during the last experiments. Correction factors were then calculated to ensure synchronization with the BH3 device.

The second part involved synchronizing sensor data with the event time markers generated by the ColEmo app. Event time markers refer to specific points in time indicated by the GUI logs, such as the start and end times of a certain stimulus (see Table 7 for an extensive list). The goal of this synchronization is to ensure that the time values associated with GUI events align correctly with the sensor data. For this part, we made a distinction between the participants who have their VAD data recorded, and those who do not. The former could be synchronized using their VAD phase data, as depicted in Fig. 3b. We first applied the same VAD algorithm to the accelerometer data that was used for the microphone data. Subsequently, all sensor data was shifted to minimize the distance between the accelerometer-detected and the microphone-detected voice onsets/offsets. To implement this, we compared our detection algorithm per participant on all axes (including magnitude of 3D acceleration) of both the Front and the Back sensors. For the synchronization purpose, we only took into account those onset/offsets that were best detected by the algorithm according to careful visual inspection. For the VAD-less group, we conducted manual synchronization taking into account two key criteria: (1) we ensured that all three jumps were completed before being indicated by their corresponding GUI time marker (*eoj* < 0, see Table 7); and (2) we verified that the walking activity for all 38 experiments fit within the corresponding time window of the GUI application, aligning to our best effort with the period between the issuance of the walking instruction and the start of the next experiment (*walkDetect*_*i*_ > *walkB*_*i*_ and *walkFinish*_*i*_ < *newExp*_*i*+1_, see Table 7).

We also identified missing data, imperfections, and anomalies. All identified problems are included in a CSV table along with the dataset (meta.csv).

## Data Records

The EmoWear dataset is available at Zenodo^65^ at the following DOI link: 10.5281/zenodo.10407278. The data is divided into three main packages: raw, mat, and csv, each serving distinct purposes to comply with various user needs and software compatibility. In addition to the mentioned packages, three separate files are included: (1) questionnaires.csv containing all participants’ responses to the pre-experiment questionnaires, (2) meta.csv containing information about data completeness (all identified missing data, imperfections, and anomalies), and (3) sample.zip containing sample data of one participant from all three provided packages. This sample file provides an easy way to check out the records in the dataset without having to download the whole data. The three packages of data are discussed below.

### raw

The raw data package contains the original, unprocessed data collected during the study. The data is organized into folders named according to a specific convention, denoted as “[code]-[ID]”, representing individual participants. Additionally, a SQLite database file (mqtt.db) is included in the package. This database file holds ColEmo logs including event time markers, self-assessment reports, and pre-experiment questionnaires of all participants. Within each participant’s folder, there are zip files corresponding to the wearable sensors, each containing raw data in the form of CSV files. An info.txt file is provided within each device’s zip file, offering detailed explanations of the raw sensor data and file contents. Please note that the column order of the CSV files in the STb devices changed starting from participant code 6 onwards, including code 6 itself. This change is reflected in the info.txt files as well.

Also, we identified and resolved two problems in the SQLite database file, mqtt.db: (1) The entry for the “vidE” event in the 19^*th*^ sequence for the participant with the ID “*9UP0*” was missing. We manually duplicated the “postB” event from the same experiment and modified it to “vidE” to solve the issue. Please note that these two markers are simultaneous (see Table 7). (2) The entry for the “participantRegistered” event for the participant with the ID “*9VYD*” was mistakenly stored in a separate database file due to a malfunction in the OneDrive synchronization mechanism. To resolve this issue, we manually transferred this missing entry to the main mqtt.db file.

### mat

The mat data package contains cleaned and synchronized participant data suitable for analysis within MATLAB. The folder structure within this package replicates the convention of the raw package, using “[code]-[ID]” folder names to distinguish individual participants. Inside each participant folder, four MAT files are found: signals, markers, surveys, and params. The signals structure provides access to synchronized wearable sensor data. It is organized as MATLAB structural variable with sensor names serving as structural fields. Each of these fields contain tables of data categorized per signal name that the wearable device provides. markers holds tables specifying time markers for crucial events during the experiments (see Table 7). markers is further divided into the “*unique*,” “*phase1*,” and “*phase2*” tables. *unique* table provides the unique time of end of jumps (*eoj*), beginning and end of the VAD phase (*vadB* and *vadE*), beginning and end of the initial baseline recording (*baselineB* and *baselineE*), and finally, beginning and end of the mid-experiments break (*pauseB* and *pauseE*). *phase1* table holds time markers of the detected beginnings (*onset*) and ends (*offset*) of the 10 sentences, read out by the participant during the first phase of the experiments i.e. the VAD phase. *phase2* contains the event time markers during the second phase of the experiments, i.e. the elicit-assess-walk sequences. surveys is a table that holds the participant’s self-assessment survey responses for all experiment sequences of the second phase. All timestamps found in the mat package are referenced with the sync moment, in which the experimenter indicated that the synchronization jumps of the participant were completed by pressing a button in ColEmo. Finally, the params table holds the time shifts and correction factors that were applied per wearable device in order to synchronize them. See Tables 6 and 7 for detailed description of mat package contents.

**Table 6:** Overview of the data records per participant in the mat and the csv packages.

mat Package			csv Package	Table Columns	Description
Variable	Field	Table	Naming Convention		
signals	e4	acc	[Variable]-[Field]-[Table].csv	timestamp [s], *x*, *y*, *z* [*mg*]	Synchronized sensor data
		bvp		timestamp [s], value [bits]	
		eda		timestamp [s], value [*µS*]	
		skt		timestamp [s], value [*°C*]	
		hr		timestamp [s], value [beats per minute]	
		ibi		timestamp [s], value [*s*]	
	bh3	acc		timestamp [s], *x*, *y*, *z* [*mg*]	
		ecg		timestamp [s], value [6.25 *µV*]	
		rsp		timestamp [s], value [bits]	
		rr		timestamp [s], value [*ms*]	
		bb		timestamp [s], value [*ms*]	
		br		timestamp [s], value [breaths per minute]	
		hr		timestamp [s], value [beats per minute]	
		hr_confidence		timestamp [s], value [%]	
	front, back, water	acc		timestamp [s], *x*_1_, *y*_1_, *z*_1_, *x*_2_, *y*_2_, *z*_2_, *x*_3_, *y*_3_, *z*_3_ [*mg*]	
		gyro		timestamp [s], *x*, *y*, *z* [*mdps*]	
markers		unique	[Variable]-[Table].csv	eoj, baselineB, baselineE, pauseB, pauseE, vadB, vadE [*s*]	Event time markers
		phase1		sentence [#], onset, offset [*s*]	
		phase2		seq, exp [#], newExp, preB, vidB, postB, surveyB, walkB, walkE, walkDetect, walkFinish [*s*]	
surveys		surveys	surveys.csv	seq, exp [#], valence, arousal, dominance, liking (1–9, 0.1 steps), familiarity (1–5, integers)	Self-assessment surveys
params		params	params.csv	device, shift [s], cf	Applied synchronization parameters

Data listed under the “Table” column of the “mat Package” can be found in the form of CSV files in the csv package. The last column lists the column contents of the tables present in both packages. Acronyms used in the table: e4—Empatica E4; bh3—BioHarness 3; acc—Accelerometer; bvp—Blood Volume Pulse; eda—Electrodermal Activity; skt—Skin Temperature; hr—Heart Rate; ibi—Inter-beat Interval; ecg—Electrocardiography; rsp—Respiration; rr—R wave to R wave; bb—Breath to Breath; br—Breath Rate; gyro—Gyroscope; params—Parameters; s—seconds; ms—milli seconds; mg—milli gee; *µS*—micro Siemens; [*°C*]—degrees Celsius; *µV*—microvolts; mdps—milli degrees per second; seq—sequence; exp [#]—experiment number; cf—correction factor.

**Table 7:** Meaning of the event time markers used in the dataset.

Event Time Marker		Meaning
raw package	mat/csv package	
participantRegistered	—	Initial interaction with GUI (“Start” pressed).
qSubmit	—	Submission of the pre-experiment questionnaire (“Submit” pressed).
signalSyncB	—	Beginning of signal synchronization jumps: waiting for wearing sensors and the jumps.
—	eoj	Moment that the magnitude of acceleration in the Zephyr BH3 crosses the gravitational value (g) for the second time after the second major peak found for the last jump (see Fig. 3). Used for markers synchronization.
signalSyncE	—	When synchronization jumps were completed (“Done” pressed). The zero reference time in mat/csv packages.
vadB	vadB	Beginning of the VAD phase. Started with asking consent for recording.
speechB	onset	Onset of a sentence detected in microphone data. Repeated 10 times.
speechE	offset	Offset of a sentence detected in microphone data. Repeated 10 times.
vadE	vadE	End of the VAD phase. Either skipped or confirmed the voice segments.
trialB	—	Begining of the trial sequence (“See the Trial Sequence” pressed).
trialE	—	End of the trial sequence (“Continue” pressed).
expsB	—	Beginning of second phase experiments after experimenter left the room (“Start Experiments” pressed). Simultaneous with “baselineB”.
baselineB	baselineB	Beginning of 2-minute baseline recording while a fixation cross is presented.
baselineE	baselineE	End of 2-minute baseline recording while a fixation cross is presented.
newExpID	newExp	A new experiment starts with showing a progress indicator. Drinking was not allowed after this moment.
preB	preB	Beginning of 5-seconds pre-elicitation fixation cross.
preE	—	End of 5-seconds pre-elicitation fixation cross. Simultaneous with “vidB”.
vidB	vidB	Beginning of emotionally stimulus video.
vidE	—	End of emotionally stimulus video. Simultaneous with “postB”.
postB	postB	Beginning of 3-seconds post-elicitation fixation cross.
postE	surveyB	End of 3-seconds post-elicitation fixation cross. Simultaneous with presentation of the self-assessment survey.
surveySubmit	—	Submission of the self-assessment survey (“Submit” pressed). Simultaneous with “walkB”.
walkB	walkB	Moment that the instruction to begin walking appears on screen.
walkE	walkE	Moment that the on-screen walking timer was over (19 seconds after “walkB”). Drinking was only allowed after this and until the next “newExp” moment.
—	walkDetect	Manually marked beginning of the participant’s actual movement. Found by visual inspection on the Zephyr BH3 acceleration data. Used for markers synchronization.
—	walkFinish	Manually marked end of the participant’s actual movement. Found by visual inspection on the Zephyr BH3 acceleration data. Used for markers synchronization.
pauseB	pauseB	Beginning of approximately 5 minutes break after the 19th sequence.
pauseE	pauseE	End of approximately 5 minutes break after the 19th sequence.
expsE	—	End of second phase experiments. Simultaneous with “walkE” of the 38th sequence.

Some excessive or duplicated markers used in the initial raw package were removed, some changed names to a more precise/descriptive name, and some new useful event times were later introduced and marked in the mat and the csv packages.

### csv

To provide compatibility with a wider range of data analysis tools, the csv data package mirrors the mat package but presents the data in CSV format. It shares the same organizational structure, with folders per participant. Each folder contains a total of 25 CSV files (6 files for E4, 8 files for BH3, 2 files × 3 STbs for front, back, and water, 3 files for markers, 1 for surveys, and 1 for synchronization parameters). The naming convention for these CSV files reflects the structure of the corresponding MATLAB variables, *e.g*., signals-bh3-ecg.csv and markers-phase1.csv. Tables 6 and 7 provide detailed information on the data records of the csv package.

## Technical Validation

We analysed technical validity of the EmoWear dataset from three aspects: (1) effectiveness of the emotion elicitation, (2) quality assessment of the collected signals using the Signal-to-Noise Ratio (SNR) metric, and (3) correlation of comparable signals collected from different body positions. We further explain these aspects in the three following subsections. The statistical analyses in this section was conducted using R v4.3.1, while the calculations for SNR and the correlation matrices were performed in MATLAB R2023b.

### Elicitation success

**Fig. 4:**
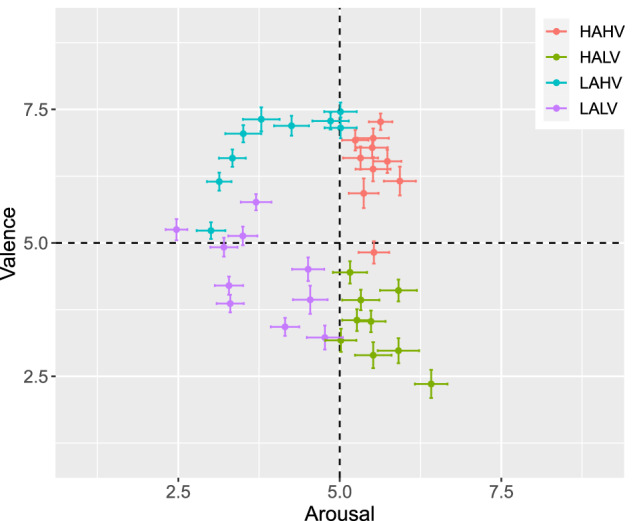
Mean locations of the self-reported affective state on the valence-arousal plane for each video clip. The colors correspond to the expected regions based on the labels from video selection procedure, and the bars indicate the standard errors for each video clip.

**Fig. 5:**
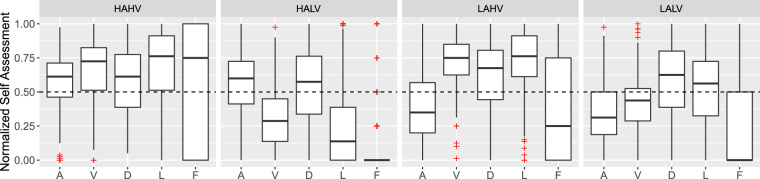
The distribution of the participants’ self-assessment scores per reported scale (A: arousal, V: valence, D: dominance, L: liking, F: familiarity) for the four different elicitation conditions (HAHV, HALV, LAHV, LALV). All scales are normalized between 0 and 1. The dotted line represents the midpoint of the scales, a threshold used to distinguish high- from low-valence/arousal in the four elicitation conditions.

As mentioned in the Methods section, different video clips were targeted at eliciting emotions in specific quadrants of the valence-arousal space (*i.e*., HAHV, HALV, LAHV, LALV; *a.k.a*. the reference labels). We compare the self-assessment scores of our participants collected in our experiments against the reference video clip labels (taken from the DEAP dataset^29^). Fig. 4 illustrates a scatter plot of the mean location of the self-assessed valence and arousal per video clips, and Fig. 5 shows distribution box plots of all the self-assessed scales per video clip conditions. The reference labels of valence-arousal quadrants in both figures (the legends in Fig. 4 and the conditions in Fig. 5) are determined by the DEAP dataset^29^.

To test whether the presented video clips elicited a variety of emotions, we used one-way repeated-measures ANalysis Of VAriance (rmANOVA)^66^ with its independent variable being the reference label. We used Mauchly’s test of sphericity to test whether or not the assumption of sphericity is met. The degrees of freedom were then adjusted using the Greenhouse–Geisser correction to account for violations of sphericity assumption. To check whether the effect sizes are substantive, we calculated the recommended effect sizes of eta-squared (*η*^2^)^67,68^. Table 8 summarizes results of the one-way rmANOVA, as well as descriptive statistics (mean and standard deviation) of the self-assessed scales per video clip condition. Results of the Figs. 4 and 5, and Table 8 suggest that presenting the video clips elicited the targeted emotions from the valence-arousal quadrants. Significant results of the rmANOVA indicate differences between video clip conditions for the scales of valence, arousal, liking, and familiarity, but not dominance. The box plots in Fig. 5 further explain the acquired significance of rmANOVA for different scales.

**Table 8:** Results of one-way repeated measures-ANalysis Of VAriance (rmANOVA) of the self-assessed scales between film clip conditions (reference labels), as well as the means and Standard Deviations (SDs) of the self-assessed scales per film clip conditions.

Self-assessed scales	Mean (SD) per film clip categories				rmANOVA			
	HAHV	HALV	LAHV	LALV	DFn	DFd	F	*η*^2^
Valence	6.44 (1.61)	3.44 (1.61)	6.82 (1.38)	4.42 (1.54)	2.15	101.51	185.20^***^	0.79
Arousal	5.53 (1.60)	5.56 (1.85)	3.99 (1.86)	3.75 (1.72)	1.85	87.35	64.60^***^	0.57
Dominance	5.80 (1.90)	5.48 (2.23)	5.99 (2.02)	5.80 (2.07)	1.87	87.90	2.22	0.40
Liking	6.48 (2.27)	2.83 (2.11)	6.84 (1.88)	5.30 (2.16)	1.98	93.36	130.10^***^	0.73
Familiarity	3.09 (1.75)	1.28 (0.72)	2.46 (1.68)	2.06 (1.47)	2.03	95.47	74.68^***^	0.61

Reported columns of the rmANOVA include: corrected degrees of freedom for the numerators and denominators (DFn and DFd respectively), using Greenhouse-Geisser correction, F-Ratios (F), and the eta-squared effect sizes (*η*^2^).

^***^p ≤ 0.001.

### Signal to noise ratios

We analyzed the raw signals obtained from wearable devices to estimate their SNRs. We used an algorithm that fits a second order polynomial to the autocorrelation function of the signal, around the *n* = 0 lag^69^. This algorithm assumes the noise to be white. While this assumption may not necessarily be accurate, it is a practical approach for SNR estimation that is also used in similar datasets^22,23^.

We estimated SNRs separately for all raw recordings in both experiment phases. For the phase 1, a single SNR was estimated per raw signal recording for each participant, representing the SNR value during the vocal vibration recording interval, *i.e*. from the onset of the first uttered sentence until the offset of the last one. In phase 2, we distinctively examined intervals during which participants were expected to remain seated (referred to as sitting intervals) and those when they were not (referred to as moving intervals). Using terms of the event time markers presented in Table 7, these intervals translate to (*newExp*_*i*_, *walkB*_*i*_) and (*walkB*_*i*_, *newExp*_*i*+1_), respectively. For the 19*th* sequence (after which a break is planned) and the last sequence (usually the 38*th* for most participants), moving interval is defined differently, *i.e.:* (*walkB*_*i*_, *walkFinish*_*i*_).

Table 9 lists descriptive statistics of the estimated SNRs. Computed values indicate high quality of the recorded signals. The mean SNRs range from 21.9 *dB* (EDA signal of the E4 device in moving intervals) to 49.8 *dB* ($\parallel \overrightarrow{AC{C}_{3}}\parallel$ signal of the Front STb in sitting intervals). The minimum SNR of the dataset is 8.9 *dB* corresponding to the EDA signal of the E4 device in one of the moving intervals; however, still 99.7% of the EDA signals during the moving intervals had SNR values above 15.1 *dB*.

**Table 9:** Descriptive statistics of the Signal-to-Noise Ratios (SNRs) computed for the recorded raw signals.

	E4				BH3			Front STb				Back STb				Water STb			
	BVP	EDA	TMP	$\parallel \overrightarrow{{\boldsymbol{A}}{\boldsymbol{C}}{\boldsymbol{C}}}\parallel$	ECG	RSP	$\parallel \overrightarrow{{\boldsymbol{A}}{\boldsymbol{C}}{\boldsymbol{C}}}\parallel$	$\parallel \overrightarrow{{\boldsymbol{A}}{\boldsymbol{C}}{{\boldsymbol{C}}}_{{\bf{1}}}}\parallel$	$\parallel \overrightarrow{{\boldsymbol{A}}{\boldsymbol{C}}{{\boldsymbol{C}}}_{{\bf{2}}}}\parallel$	$\parallel \overrightarrow{{\boldsymbol{A}}{\boldsymbol{C}}{{\boldsymbol{C}}}_{{\bf{3}}}}\parallel$	$\parallel \overrightarrow{{\boldsymbol{G}}{\boldsymbol{Y}}{\boldsymbol{R}}{\boldsymbol{O}}}\parallel$	$\parallel \overrightarrow{{\boldsymbol{A}}{\boldsymbol{C}}{{\boldsymbol{C}}}_{{\bf{1}}}}\parallel$	$\parallel \overrightarrow{{\boldsymbol{A}}{\boldsymbol{C}}{{\boldsymbol{C}}}_{{\bf{2}}}}\parallel$	$\parallel \overrightarrow{{\boldsymbol{A}}{\boldsymbol{C}}{{\boldsymbol{C}}}_{{\bf{3}}}}\parallel$	$\parallel \overrightarrow{{\boldsymbol{G}}{\boldsymbol{Y}}{\boldsymbol{R}}{\boldsymbol{O}}}\parallel$	$\parallel \overrightarrow{{\boldsymbol{A}}{\boldsymbol{C}}{{\boldsymbol{C}}}_{{\bf{1}}}}\parallel$	$\parallel \overrightarrow{{\boldsymbol{A}}{\boldsymbol{C}}{{\boldsymbol{C}}}_{{\bf{2}}}}\parallel$	$\parallel \overrightarrow{{\boldsymbol{A}}{\boldsymbol{C}}{{\boldsymbol{C}}}_{{\bf{3}}}}\parallel$	$\parallel \overrightarrow{{\boldsymbol{G}}{\boldsymbol{Y}}{\boldsymbol{R}}{\boldsymbol{O}}}\parallel$
Sitting intervals (vocal vibration recording interval included)																			
Count	1798	1798	1798	1798	1798	1798	1798	1703	1703	1703	1703	1637	1637	1637	1637	819	819	819	819
Mean	27.8	27.1	27.2	33.4	22.6	35.2	39.0	49.0	49.6	49.8	35.1	48.0	48.4	48.5	36.9	48.0	48.4	48.6	32.1
SD	2.6	1.0	0.4	2.5	4.0	0.4	0.7	0.5	0.6	0.5	3.4	0.6	0.7	0.6	3.6	0.8	0.8	0.5	3.2
Min	13.9	14.4	25.2	20.1	15.7	33.2	36.5	45.4	43.4	46.4	24.5	43.1	41.6	45.0	16.7	35.9	36.9	46.4	16.6
*Q*_0.3_	17.7	20.7	25.2	23.7	15.8	33.2	36.6	46.9	47.1	47.5	25.8	45.0	45.0	45.7	25.8	41.4	42.0	46.5	20.6
*Q*_25_	26.6	26.8	27.0	31.9	20.1	35.0	38.8	48.8	49.4	49.5	32.9	47.8	48.2	48.3	34.8	47.9	48.2	48.3	30.4
*Q*_50_	27.9	27.2	27.2	34.1	21.9	35.2	39.1	49.1	49.6	49.8	35.4	48.0	48.5	48.5	37.1	48.0	48.5	48.5	33.2
*Q*_75_	29.3	27.5	27.4	35.4	24.0	35.4	39.4	49.3	49.9	50.0	37.3	48.3	48.8	48.8	39.3	48.3	48.7	48.8	34.3
Max	37.7	32.2	30.9	37.6	45.5	38.8	40.8	51.5	52.4	52.5	45.3	50.9	51.9	51.5	48.3	50.3	51.0	51.2	40.6
Moving intervals																			
Count	1776	1776	1776	1776	1776	1776	1776	1681	1681	1681	1681	1615	1615	1615	1615	798	798	798	798
Mean	27.0	21.9	22.1	22.8	25.1	30.1	34.1	44.1	44.1	43.5	43.0	42.8	42.8	41.8	41.7	42.8	43.0	43.4	29.6
SD	2.3	1.4	1.0	2.0	6.2	1.0	0.8	1.0	1.2	1.1	2.0	1.2	1.3	1.5	2.9	1.3	1.2	1.0	5.5
Min	16.2	8.9	20.2	16.0	15.0	28.3	28.9	39.6	37.4	39.2	34.8	34.2	33.1	36.8	30.7	34.9	34.7	41.1	9.1
*Q*_0.3_	19.0	15.1	20.5	17.3	15.3	28.6	31.7	41.5	41.0	40.3	37.6	36.2	35.3	37.1	31.4	35.0	36.0	41.4	17.6
*Q*_25_	25.8	21.3	21.5	21.5	20.8	29.5	33.7	43.5	43.4	42.9	41.7	42.2	42.2	41.1	40.2	42.4	42.5	42.8	25.7
*Q*_50_	27.1	21.8	21.9	22.8	23.3	30.0	34.1	44.1	44.1	43.4	43.3	42.8	42.8	41.9	42.2	42.9	43.0	43.3	29.9
*Q*_75_	28.5	22.6	22.6	24.3	27.6	30.6	34.6	44.7	44.7	44.0	44.6	43.4	43.5	42.7	43.7	43.5	43.6	43.9	33.2
Max	36.8	35.8	35.8	29.7	46.5	43.7	41.4	54.9	57.3	57.1	47.1	54.0	56.3	56.3	47.8	47.4	47.8	49.3	44.3

The columns correspond to signal names listed in Table 2. For the ACC and GYRO signals, SNRs are calculated for the norm of acceleration and rotation vectors. All values are expressed in decibels (dB) except for count. *Q*_*x*_ represents quantile values where *x* indicates the percentage of SNR samples falling into the bin from the minimum to *Q*_*x*_.

### Sensor correlations

To further evaluate technical validity of our dataset, we investigate correlations between sensor data collected from our different sensors used, as recommended in^49^. A look at our available sensor data suggests that we have three sets of comparable data collected from different body sources: a) the heartbeat set, referring to heartbeat-related data including BVP from the E4 device, ECG from the BH3 device, and chest/back vibrations from the Front/Back STbs. b) the breathing set, referring to breathing-related data including RSP from the BH3 device and chest/back vibrations from the Front/Back STbs. c) The motion set, referring to pure movement data from all IMUs (E4, BH3, Front/Back STbs). For SCG signal within the heartbeat and breathing sets, we chose the dorsoventral (z) and the mediolateral (x) axes of the Front/Back ACC and GYRO signals respectively. These axes are expected to be the most promising for this type of analysis in the literature^70,71^.

The heartbeat set and the breathing set involve signals with distinct morphological properties within the sets. To conduct a meaningful comparison through correlation analysis, we pre-processed those signals to amplify those properties expected to remain similar across diverse signal types. For example, in the case of the heartbeat set, our pre-processing focused on accentuating variations originating from the heartbeats rather than emphasizing their unique morphological properties (such as the QRS complex, the well-known morphological properties of an ECG signal). This was achieved by filtering the signals in bandpasses of interest (0.67 to 3.33 Hz for the heart set and 0.05 to 0.7 Hz for the breathing set) and extracting their autocorrelation sequences. Subsequently, Pearson correlation coefficients were calculated within each set and averaged over the whole dataset.

**Fig. 6:**
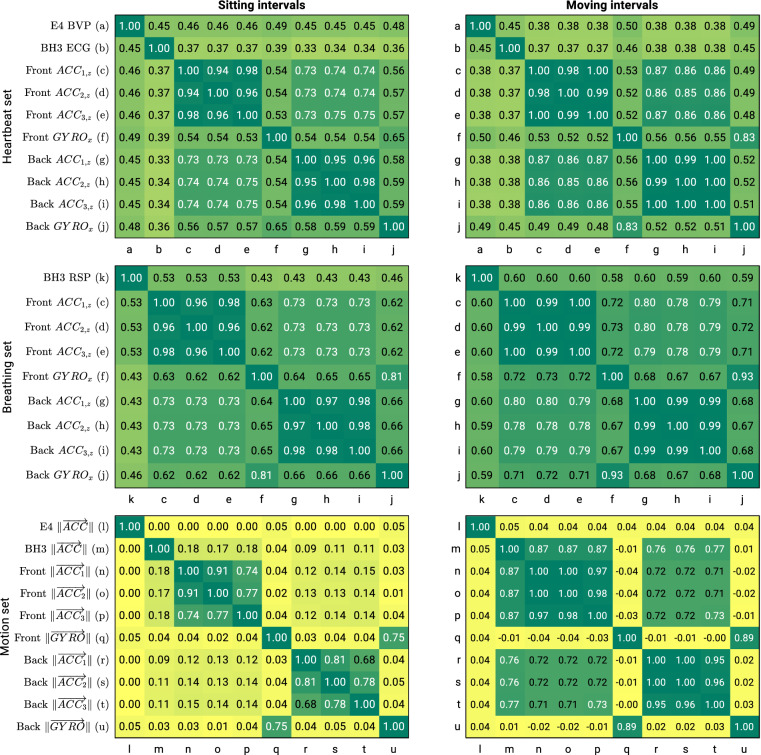
Pearson correlation heat maps, computed separately for sitting intervals (from the beginning of progress indicator presentation to self-assessment form submission) and moving intervals (from self-assessment form submission to the beginning of the progress indicator for the subsequent experiment) and averaged over the entire dataset. Compared signals (referred to as listed in Table 2) are noted in the matrix rows and are assigned letters that are reused wherever the same source is used. *Note 1:* Signals from the heartbeat set and the breathing set undergo preprocessing using bandpass filters and the calculation of their autocorrelation sequences. *Note 2:* Filter cut-offs applied to the heartbeat set and the breathing set differ, even though identical letters may be found.

Fig. 6 presents the correlation results using heatmaps. Whenever the average correlation is above 0.3, all the p-values are below 0.05. Results suggest consistent and meaningful relationships among the measurements taken from the compared sources. Note that the amplitudes of accelerations recorded from the wrist (E4) show no correlation with those recorded from the trunk (BH3 and Front/Back STbs). This lack of correlation can be explained by the natural different movements of the wrist compared to those of the trunk. Similarly, little correlations are observed among the BH3, Front, and Back STbs during sitting intervals when they only measure small vibrations from different positions on the trunk. However, these correlations increase during moving intervals, highlighting the dominance of similarly sensed motions of different body trunk positions during gaits. In summary, the correlation results suggest: (1) absence of systematic malfunctioning in the sensor measurements, as the correlations align with expectations, (2) feasibility of conducting HRV and breathing analysis through the measured inertial data, as they demonstrate meaningful correlation with BVP and ECG data.

## Usage Notes

### Data interpretation

The three accelerometers and the gyroscope of the ST STb devices were initially configured to provide output data rates as listed in Table 2. However, their data was written to the output files using the task scheduler of the FreeRTOS^72^ within the STbs firmware. As a result, the timestamps of these sensors are irregular, which must be considered when working with their data. To convert these irregular timestamps to regular ones, functions like interp1 from the MATLAB Signal Processing Toolbox and interp from the Python Numpy library are useful.

For guidance on performing emotion recognition, we direct readers to a comprehensive review of the common steps required for emotion recognition from wearable physiological signals^12^. For available methods on the use of chest-worn accelerometer data for VAD, activity recognition, drinking detection, etc., we direct readers to our comprehensive survey on applications and methods associated with chest-worn IMUs^21^.

### Limitations

As detailed in the Methods section, the synchronization of event markers with sensor data needed manual intervention. This need arose from an incorrect assumption of ours concerning the time synchrony between the two laptops used in the experiments, namely the experimenter’s and the participant’s laptops. The Zephyr BH3 devices were consistently synchronized with the experimenter’s laptop, as data was copied from and the device was recharged through this laptop. However, the event time markers were generated by the participant’s laptop, which was responsible for running the ColEmo GUI. The synchrony among these two laptops was neither sufficient nor consistent throughout the data collection period, for synchronization of the event markers with the BH3 device.

Another limitation was lack of VAD and the drinking data for the first 26 participants as well as the lack of a pause for the first 2 participants. The reason was that the ColEmo GUI faced two updates throughout the data collection period. First, happened after the 2^*nd*^ participant. In this update, the 5-minute break was added in the middle of experiments based on the feedback by the first two participants. Also, the sequence number which was not sent along with the MQTT log messages was added to the logs. Second update took place after the 26^*th*^ participant. In this update, we added VAD phase in the beginning and started using the third STb device on participants’ cup of water. Therefore, the first 26 participants are missing the VAD and the drinking data (codes 1–26). Furthermore, due to a technical issue, there is an absence of drinking data for participant 34, and due to an unintended failure to run the logging software, time markers from participant 35 were not recorded, making their sensor data incomprehensible. Details regarding such instances of data incompleteness and all identified problems are documented in a meta file available in.csv format, along with the data.

### Accessibility

The EmoWear dataset is fully accessible to the public without restriction at: 10.5281/zenodo.10407278. However, in compliance with GDPR regulations, we strictly prohibit any attempt by the dataset users to reveal and/or disclose identity or personal information of the participants.

## Data Availability

We used ColEmo, our open-source GUI for emotion data collection in this study. The code base of ColEmo is available at https://gitlab.ilabt.imec.be/emowear/colemo with its architecture and usage framework explained in^55^. The code used for data cleaning, synchronization, and technical validation are publicly available at https://gitlab.ilabt.imec.be/emowear/preprocessing.
